# High-dose stereotactic body radiotherapy using CyberKnife® for stage I peripheral lung cancer: a single-center retrospective study

**DOI:** 10.1186/s13014-022-02094-3

**Published:** 2022-07-19

**Authors:** Yasuhiro Ryuno, Takanori Abe, Misaki Iino, Satoshi Saito, Tomomi Aoshika, Tomohiro Oota, Mitsunobu Igari, Ryuta Hirai, Yu Kumazaki, Kyoichi Kaira, Hiroshi Kagamu, Hironori Ishida, Shin-ei Noda, Shingo Kato

**Affiliations:** 1 Department of Radiation Oncology, International Medical Center, Saitama Medical University, 1397-1, Yamane, Hidaka, Saitama 350-1298 Japan; 2 Department of Respiratory Medicine, International Medical Center, Saitama Medical University, Hidaka, Japan; 3 Department of General Thoracic Surgery, International Medical Center, Saitama Medical University, Hidaka, Japan

**Keywords:** Early-stage lung cancer, CyberKnife, Stereotactic body radiotherapy, Local control, Radiation pneumonitis

## Abstract

**Background:**

This retrospective study was performed to evaluate the efficacy and toxicity of high-dose stereotactic body radiotherapy (SBRT) using a CyberKnife® for patients with stage I peripheral non-small cell lung cancer (NSCLC).

**Methods:**

Ninety-six patients with stage I peripheral NSCLC who were treated with SBRT using a CyberKnife® from August 2010 to June 2019 were identified and included in this study. Local control (LC), local progression-free survival (LPFS), progression-free survival (PFS), overall survival (OS), and late toxicity were evaluated. Potential risk factors associated with LC, LPFS, PFS, or OS were investigated by univariate analyses.

**Results:**

Data of 96 patients were examined. The prescribed dose to the tumor was 54 Gy in 3 fractions in 91 patients and 60 Gy in 3 fractions in 5 patients. The median follow-up duration was 27 months. The 2-year LC, LPFS, PFS, and OS rates were 97%, 88%, 84%, and 90%, respectively. The T factor was significantly correlated with LC, LPFS, and PFS. The 2-year LC rate for patients with T1a/T1b and T1c/T2a disease was 100% and 90%, respectively (*p* < 0.05), and the 2-year PFS rate for the corresponding patients was 95% and 65%, respectively (*p* < 0.001). One patient (1%) developed grade 3 radiation pneumonitis.

**Conclusions:**

High-dose SBRT using a CyberKnife® for stage I peripheral NSCLC produced favorable treatment outcomes with acceptable late toxicity. Further studies are needed to improve the treatment outcomes for patients with T1c/T2a disease.

## Background

Lung cancer is the leading cause of cancer mortality worldwide [1, 2]. Surgery is the gold standard treatment for early-stage non-small cell lung cancer (NSCLC) [3]. However, some patients may be unable to tolerate surgery because of old age, poor organ function, and/or concomitant illnesses. Stereotactic body radiotherapy (SBRT) is a high-precision radiotherapy technique that uses multiple beams from several directions to target a solitary tumor, enabling administration of a very high radiation dose to the tumor while minimizing doses to the surrounding normal tissues. Based on the results of several clinical studies conducted in Japan and North America [4–6], SBRT using a high-dose hypofractionation regimen is a viable option for patients with inoperable early-stage peripheral NSCLC [3].

Various types of radiotherapy machines and radiotherapy delivery techniques of SBRT for lung tumors have recently been developed. Linear accelerator-based SBRT (Linac-SBRT) is used worldwide and has produced favorable treatment results for early-stage NSCLC [4–7]. The CyberKnife® (CK) (Accuray, Sunnyvale, CA, USA) is another specialized machine for delivering robotic stereotactic radiotherapy that allows highly conformal irradiation with tumor motion tracking [8]. Several reports have described the efficacy and safety of CK-based SBRT (CK-SBRT) for early-stage NSCLC [9–12].

Although several studies of SBRT for early-stage NSCLC have shown promising treatment results, these studies used various dose-fractionation schedules. Several multi-institutional prospective studies of Linac-SBRT adopted a dose of 48 Gy in four fractions or 54 Gy in three fractions [4–6], and several CK-SBRT studies used a dose of 45, 54, or 60 Gy in three fractions [9–12]. Therefore, the optimal dose-fractionation regimen to maximize tumor control and minimize toxicity has not been determined.

Although SBRT can reduce the normal lung volume irradiated with high-dose versus conventional three-dimensional conformal radiotherapy, a high radiation dose with a hypofractionated schedule may cause severe lung toxicity. CK-SBRT may minimize the normal lung volume irradiated with high-dose versus Linac-SBRT [13], which may allow for safer radiation delivery to the lung. For these reasons, we have treated patients with early-stage peripheral NSCLC using CK-SBRT with 54 or 60 Gy in three fractions. The present study was performed to evaluate the efficacy and safety of this treatment and investigate the risk factors for recurrence.

## Methods

### Patients

The study cohort comprised patients with NSCLC treated with SBRT alone using a CK at our institute. The inclusion criteria were (1) stage I NSCLC according to the eighth version of the Union for International Cancer Control staging system, (2) a European Cooperative Oncology Group performance status of 0 or 1, (3) either pathologically or clinically diagnosed NSCLC, and (4) medically inoperable disease or patient refusal to undergo surgery. All patients underwent computed tomography (CT) scans of the chest and abdomen, ^18^F-fluorodeoxyglucose positron emission tomography, and magnetic resonance imaging of the brain prior to treatment. Bronchoscopy and biopsy/cytology of the tumor were performed when feasible. When bronchoscopy was not feasible because of poor respiratory/cardiac function and/or patient refusal, respiratory oncologists made a clinical diagnosis based on the progression of the radiological findings. The indications for SBRT were determined at multidisciplinary conferences comprising respiratory surgeons, medical oncologists, diagnostic radiologists, and radiation oncologists. Patients with central lung cancer, which was defined as a lesion within 2 cm of the main bronchus and/or within 1 cm of the heart, great vessels, esophagus, trachea, or brachial plexus, were excluded from the study. Patients with active interstitial pneumonia, serious comorbidities, or active double cancer were also excluded. This study was approved by our institutional review board (No. 18-132).

### Radiotherapy

Patients underwent CT scans at 1.25-mm thickness using a LightSpeed Xtra® (GE Healthcare, Chicago, IL, USA). An Esform Vacuum Bag® (Engineering System Co., Ltd., Nagano, Japan) was used for patient immobilization. Four-dimensional chest CT scans at 2.5-mm thickness were performed to evaluate tumor respiratory motion. When the tumor respiratory motion was greater than 10 mm, two or three fiducial markers (Gold Marker®; Olympus, Tokyo, Japan) were placed near the tumor with a bronchoscope and four-dimensional CT.

The gross tumor volume (GTV) was defined as the visible tumor in the lung window on the treatment planning CT images, and the clinical target volume was defined as the same volume. For patients whose tumor respiratory motion was less than 10 mm, the internal target volume (ITV) was created with maximum intensity projection methods to cover the tumor in all respiratory phases. For patients whose tumor respiratory motion was greater than 10 mm, four-dimensional CT was performed using a 320-multidetector CT scanner (Aquilion ONE®; Canon Medical Systems, Otawara, Japan), and the ITV margin to compensate for the motion error between the GTV and the fiducial marker was added to the GTV. The planning target volume was determined by adding a 2-mm safety margin in all directions to the ITV. The organs at risk (brachial plexus, trachea and main bronchus, esophagus, heart and great vessels, lung fields, ribs, and spinal cord) were contoured on the planning CT images using the soft tissue window. Treatment plans were calculated using a ray-tracing algorithm until 2015 and a Monte Carlo algorithm after 2015. A total of 54–60 Gy in three fractions was prescribed to 70–80% isodose line encompassing 99% of GTV and 95% of PTV. Theoretical basis of prescription to GTV was described in the study from Miura et al. [14].

SBRT was delivered with 6-MV X-rays using CK G3™ technology with a fixed cone. When the tumor respiratory motion was less than 10 mm, the Xsight Spine Tracking System was used. This allowed the position of the tumor to be assessed and corrected on the basis of its location relative to the spine. When the tumor respiratory motion was greater than 10 mm, the Synchrony respiratory tracking system was used, meaning that the radiation beams were moved synchronously with the fiducial markers to match the movement of the lung tumor. We did not have the Synchrony Xsight Lung Tracking System (the direct lung tumor tracking system) in our CK system. Dose constraints for organs at risk were as follows: normal lung volume receiving more than 20 Gy (V_20Gy_), < 15%; maximum dose (D_max_) to the spinal cord, < 21.9 Gy; D_max_ to the heart, < 30 Gy; minimum dose to the most irradiated 15 cm^3^ of the heart, < 24 Gy; D_max_ to the great vessels, < 45 Gy; D_max_ to the bronchus, < 23.1 Gy; and D_max_ to the trachea, < 30 Gy.

### Follow-up

After completing the treatment, all patients were followed up every 1 to 3 months for the first year and every 4 to 6 months thereafter. All patients underwent a physical examination, blood tests including measurement of serum carcinoembryonic antigen and/or squamous cell carcinoma-related antigen, chest radiographs, CT scans of the chest and abdomen, and brain magnetic resonance imaging to evaluate the disease status and late toxicities. When recurrence was suspected, ^18^F-fluorodeoxyglucose positron emission tomography and, if feasible, biopsy/cytology were performed to determine the disease status. Treatment failures were classified as local recurrence, pulmonary hilar and/or mediastinal lymph node metastasis, or distant metastasis. Late toxicities were graded in accordance with the National Cancer Institute Common Toxicity Criteria for Adverse Events version 4.0 [15].

### Statistical analysis

Local control (LC) was defined as the interval between the date of initiation of therapy and the date of recurrence of the lung tumor or the most recent follow-up. Local progression–free survival (LPFS) was defined as the interval between the date of initiation of therapy and the date of recurrence of the lung tumor, death, or the most recent follow-up. Progression-free survival (PFS) was defined as the interval between the date of initiation of therapy and the date of disease progression, death, or the most recent follow-up. Overall survival (OS) was defined as the interval between the date of initiation of therapy and the date of death from any cause or the most recent follow-up. The actuarial LC, LPFS, PFS, and OS rates were calculated with the Kaplan–Meier method.

Potential risk factors associated with LC, LPFS, PFS, and OS were investigated by univariate analyses. Age, sex, T factor, history of pulmonary surgery, histological diagnosis, operability, and comorbidities were considered binary variables. Differences between two groups were compared using the log-rank test. A *p* value of < 0.05 was considered statistically significant. All statistical analyses were performed using IBM SPSS Statistics for Windows, version 25.0 (IBM Corp., Armonk, NY, USA).

## Results

**Fig. 1:**
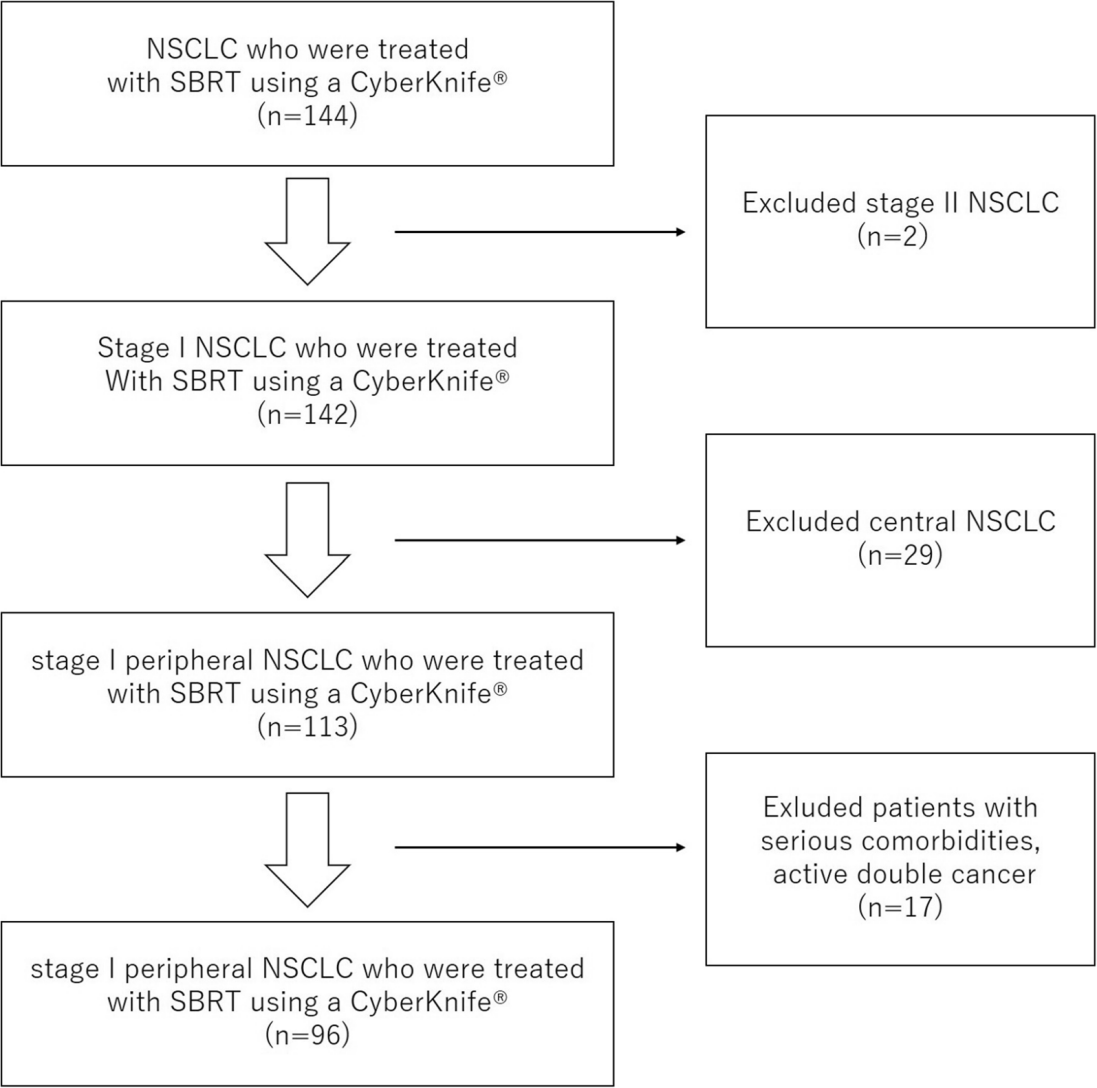
CONSORT diagram. In total, 144 patients with stage I and II NSCLC were treated with SBRT using a CyberKnife® from August 2010 to June 2019. Patients with stage II disease or central lung cancer and those who had serious comorbidities or active double cancer were excluded from the study. Finally, 96 patients with stage I peripheral NSCLC were analyzed in the current study. Abbreviations: NSCLC, non-small cell lung cancer; SBRT, stereotactic body radiotherapy

Ninety-six patients with stage I NSCLC treated from August 2010 to June 2019 were identified and included in this study (Fig. 1). Nineteen patients were histologically diagnosed, and 77 patients were clinically diagnosed. The T stage distribution was as follows: T1a, 14 patients; T1b, 47; T1c, 30; and T2a, 5. Ninety-one patients received 54 Gy in three fractions, and the remaining five patients received 60 Gy in three fractions. Median prescribed isodose line was 76% (60–96%). Median GTV D99% was 101% (83–119%) of prescribed dose and median PTVD95% was 96% (80–113%) of prescribed dose (Table 1).

**Table 1:** Patient and treatment characteristics

Characteristic	
Age, years, median (range)	77 (39–93)
Sex, n (%)	
Male	61 (64)
Female	35 (36)
Operability, n (%)	
Operable	25 (26)
Inoperable	71 (74)
Histological type, n (%)	
Adenocarcinoma	15 (16)
Squamous cell carcinoma	4 (4)
Not identified	77 (80)
T factor, n (%)	
T1a	14 (15)
T1b	47 (49)
T1c	30 (31)
T2a	5 (5)
Dose prescription to GTV, n (%)	
54 Gy in 3 fractions	91 (95)
60 Gy in 3 fractions	5 (5)
Prescribed Isodose line, %, median (range)	76 (60–97)
V_20Gy_, %, median (range)	2.4 (0.2–11.0)
GTV D_99_, %, median (range)	101.4 (82.7–118.7)
PTV D_95_, %, median (range))	95.6 (79.8–112.9)

Data are presented as median (range) or n (%). *GTV* gross tumor volume, *PTV* planning target volume, *V*_*20Gy*_ normal lung volume receiving more than 20 Gy

**Fig. 2:**
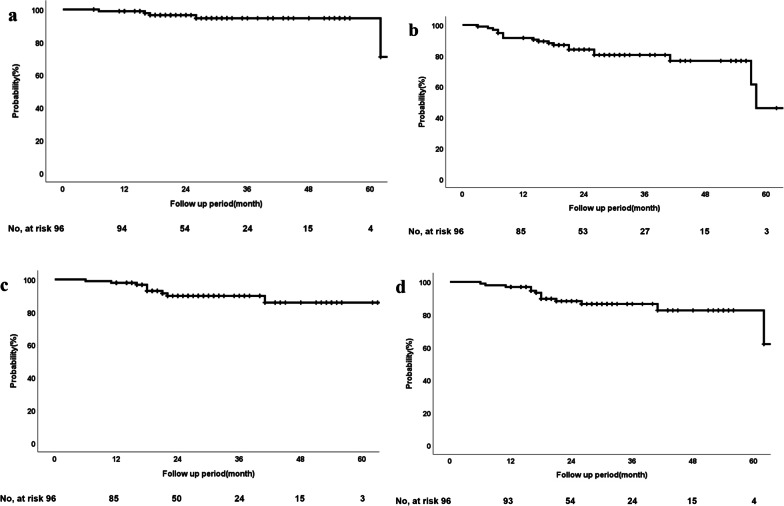
Treatment evaluation. **a** LC, **b** LPFS, **c** PFS, and **d** OS of the whole cohort (n = 96). Abbreviations: LC, local control; LPFS, local progression free survival; PFS, progression-free survival; OS, overall survival; CI, confidence interval

The duration of follow-up was 6 to 120 months (median, 27 months). During the follow-up period, 5 patients developed local recurrence; 6 developed regional lymph node metastases, including 2 hilar lymph nodes, 5 mediastinal lymph nodes, and 1 subclavian lymph node; and 11 developed distant metastases, including the lung, pleura, brain, bone, or adrenal gland. Most recurrences were observed in patients with T1c-T2 tumors (Table 2). At the last follow-up, six patients had died of lung cancer and four of other diseases. The 2- and 3-year rates of LC, LPFS, PFS, and OS for all patients were 97% (95% confidence interval [CI] 93–100%) and 95% (95% CI 89–100%), 88% (95% CI 81–95%) and 86% (95% CI 79–94%), 84% (95% CI 76–92%) and 80% (95% CI 72–89%), and 90% (95% CI 83–97%) and 90% (95% CI 83–97%), respectively (Fig. 2a–d).

**Table 2:** Patterns of failure

T factor (n)	Local recurrence	Regional recurrence	Distant metastasis
T1a/b (61)	1	1	4
T1c/T2a (35)	4	5	7
Total (96)	5	6	11

**Fig. 3:**
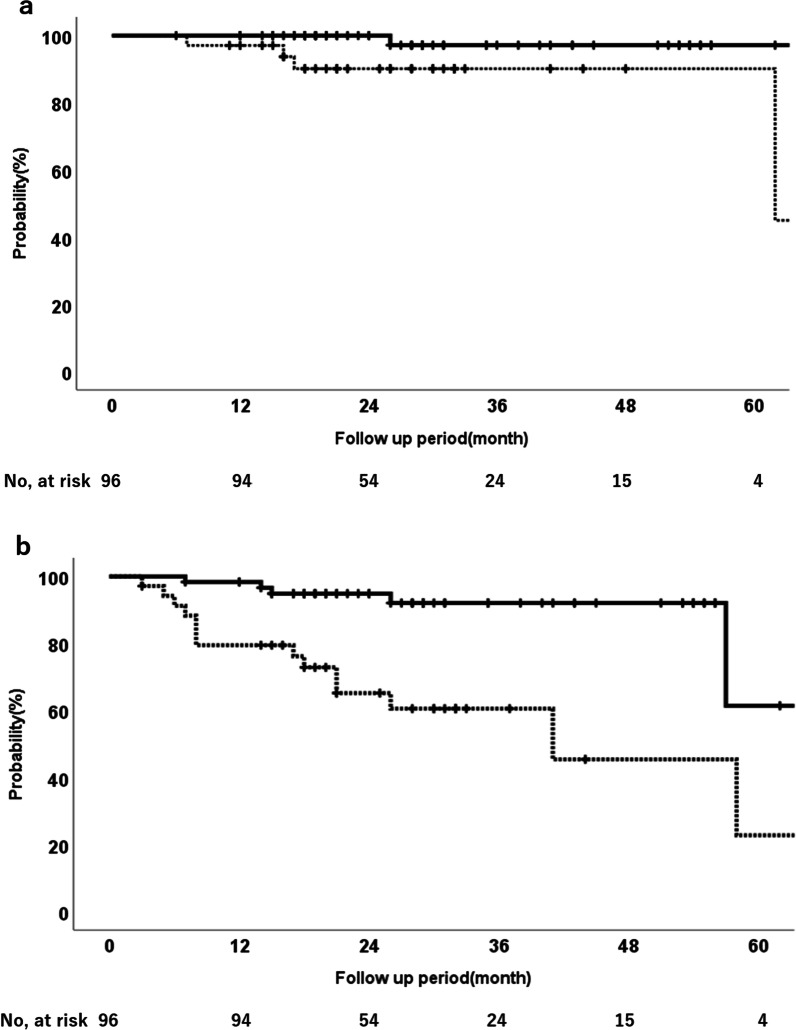
LC and PFS of the T1a/T1b and T1c/T2a groups. **a** LC of the T1a/T1b and T1c/T2a groups (solid line, T1a/T1b group; dotted line, T1c/T2b group). **b** PFS of the T1a/T1b and T1c/T2a groups (solid line, T1a/T1b group; dotted line, T1c/T2b group). Abbreviations: LC, local control; PFS, progression-free survival

The univariate analysis showed that the T factor had a statistically significant impact on LC, PFS, LPFS, and OS (Table 3). The 2-year LC rate for patients with T1a/T1b and T1c/T2a disease was 100% and 90%, respectively (*p* < 0.05), and the 2-year PFS rate was 95% and 65%, respectively (*p* < 0.001) (Fig. 3a, b). Histological confirmation also showed a significant impact on LPFS and PFS outcomes, and patients without a histological diagnosis had better LPFS and PFS than those with a histological diagnosis (Table 3).

**Table 3:** Results of univariate analysis for LC, PFS, OS, and LPFS

Characteristics	Patients (n)	2-year LC (%)	*p* value	2-year PFS (%)	*p* value	2-year OS (%)	*p* value	2-year LPFS (%)	*p* value
Age (years)									
> 75	45	97	0.822	83	0.793	85	0.454	86	0.663
≤ 75	51	96		84		94		90	
Sex									
Male	61	95	0.815	75	0.052	86	0.353	84	0.684
Female	35	100		100		97		97	
T classification									
T1a/T1b	61	100	0.044	95	< 0.001	98	< 0.001	98	< 0.001
T1c/T2a	35	96		65		76		72	
History of lung surgery									
Yes	29	96	0.939	82	0.883	88	0.689	85	0.841
No	67	97		85		91		90	
History of ischemic heart disease									
Yes	24	100	0.128	92	0.402	90	0.920	90	0.497
No	72	95		82		90		87	
Histological diagnosis									
Yes	21	88	0.277	67	0.002	89	0.123	79	0.049
No	75	90		88		98		91	
Operability									
Operable	25	100	0.226	85	0.786	96	0.747	96	0.411
Inoperable	71	95		83		88		86	

*LC* local control, *PFS* progression-free survival, *OS* overall survival, *LPFS* local progression-free survival

Regarding late toxicities, radiation pneumonitis occurred in 74 patients (77%), including grade 1 in 68 patients (71%), grade 2 in 5 patients (5%), and grade 3 in 1 patient (1%). The median interval between SBRT and the onset of radiation pneumonitis was 3 months (range 0–9 months). The patient who developed grade 3 radiation pneumonitis had simultaneous bacterial pneumonia, which may have worsened the pulmonary toxicity. No other grade 2 or higher late adverse events were observed.

## Discussion

The efficacy and toxicity of CK-SBRT for stage I peripheral NSCLC were retrospectively evaluated. In this study, a 54–60 Gy in three fractions was prescribed to 76% (60–96) isodose line encompassing 99% of GTV. The 2- and 3-year LC rates for all patients were 97% and 95%, respectively. The corresponding OS rates were 90% and 90%, respectively (Fig. 2a, d).

In SBRT for early-stage NSCLC, various dose and fractionation schedules have been used, and the optimal dose-fractionation regimen to maximize tumor control and minimize toxicity has not yet been determined. To compare different dose-fractionation regimens, radiation doses are converted to biologically effective doses (BEDs) by the linear quadratic model using an alpha/beta ratio of 10 Gy for tumors [16]. The BEDs of 48 Gy in four fractions, 54 Gy in three fractions, and 60 Gy in three fractions are 105 Gy_10_, 151 Gy_10_, and 180 Gy_10_, respectively.

Several clinical studies have focused on SBRT for early-stage NSCLC using Linac-SBRT. In the JCOG0403 study, patients were treated with a dose of 48 Gy in four fractions at the isocenter of the tumor. The study demonstrated 3-year LC and OS rates of 87.3% and 59.9%, respectively, for patients with inoperable tumors and 85.4% and 76.5%, respectively, for patients with operable tumors [5]. In the RTOG0236 and 0618 studies, patients were delivered a dose of 54 Gy in three fractions to 95% of the planning target volume. The 3-year LC and OS rates were 97.6% and 55.8%, respectively, for patients with inoperable tumors (RTOG0236) [4]. The 4-year corresponding rates were 96% and 56%, respectively, for patients with operable tumors (RTOG0618) [6]. Several CK-SBRT studies used a dose of 45, 54, or 60 Gy in three fractions and reported that the 2- or 3-year LC and OS rates were 78% to 96% and 37% to 87%, respectively (Table 4). These reports suggest that the current study yielded favorable treatment outcomes comparable to or somewhat superior to those in the other studies, although our patient population included both patients with medically inoperable tumors and those who refused to undergo surgery.

**Table 4:** Studies involving Linac-SBRT and CK-SBRT for early-stage NSCLC

	T factor (n)	LC (%)	OS (%)	Dose	Prescription	Lung toxicity
Linac-SBRT						
Timmerman et al. [4]	T1 (44)	3-year: 97.6	3-year: 55.8	54 Gy/3 Fr	PTV D_95_	Grade 3: 4%
	T2 (11)					
Nagata et al. [5]	T1 (169)	Operable	Operable	48 Gy/4 Fr	Isocenter	Operable
		3-year: 85.4	3-year: 76.5			Grade 3: 3%
		Inoperable	Inoperable			Inoperable
		3-year: 87.3	3-year: 59.9			Grade 3: 8%
						Grade 4: 1%
Koshy et al. [21]	T1 (334)	NA	3-year: 50	48–60 Gy/3–4 Fr	NA	NA
	T2 (164)		5-year: 30			
Matsuo et al. [25]	T1 (73)	3-year: 86.8	3-year: 58.6	48 Gy/4 Fr	Isocenter	Grade 3: 3%
	T2 (28)		5-year: 46.7			
CK-SBRT						
Bahig et al. [10]	T1 (123)	2-year: 96	2-year: 87	40–60 Gy/3–5 Fr	PTV D_95_	Grade 3: 1%
	T2 (27)				75% isodose	Grade 5: 2% (with IPF)
Heal et al. [11]	T1 (63)	2-year: 94	3-year: 37	50–60 Gy/3–5 Fr	NA	Grade 3: 2%
	T2 (37)	3-year: 84				
van der Voort van Zyp et al. [12]	T1 (39)	2-year	1-year: 83	45 or 60 Gy/3 Fr	PTV D_95_	Grade 3: 4%
	T2 (31)	45 Gy: 78	2-year: 62		70–85% isodose	
		60 Gy: 96				
Present study	T1a/b (61)	2-year: 97	2-year: 90	54–60 Gy/3 Fr	GTV D_99_	Grade 3: 1%
	T1c (30)				70–80% isodose	
	T2 (5)					

*Linac-SBRT* linear accelerator-based stereotactic body radiotherapy, *CK-SBRT* CyberKnife®-based stereotactic body radiotherapy, *NSCLC* non-small cell lung carcinoma, *PTV* planning target volume, *NA* not available, *IPF* idiopathic pulmonary fibrosis, *GTV* gross tumor volume, *Fr* fractions

Regarding the dose–response relationship in SBRT for early-stage NSCLC, several studies have demonstrated that a BED of ≥ 100 Gy_10_ delivered at the isocenter of the tumor produced better LC and OS outcomes than a BED of < 100 Gy_10_ [17, 18]. However, whether a higher BED (100–150 Gy_10_ or ≥ 150 Gy_10_) may contribute to better LC outcomes than a BED of 100 Gy_10_ remains controversial. In their meta-analysis involving 2587 patients with stage I NSCLC, Zhang et al. [19] reported no significant relationship for LC among patients who received a low dose (< 83.2 Gy_10_), medium dose (83.2–106 Gy_10_), medium to high dose (106–146 Gy_10_), and high dose (> 146 Gy_10_). In contrast, Mehta et al. [18] analyzed the relationship between LC and BED in a systematic review and reported a positive dose–response relationship beyond a BED of 100 Gy_10_, and 90% LC probability was achieved with a BED of > 159 Gy_10_. More recently, Stephans et al. [20] reported that a BED of 150 to 180 Gy_10_ was associated with a lower rate of local failure than a BED of 100 Gy_10_. From these results and the findings of the current study, additional randomized trials comparing SBRT regimens with BEDs ranging from 100 to 180 Gy_10_ may be needed to determine the optimum dose to maximize the LC probability.

In the current study, the T factor was a significant factor impacting LC, LPFS, PFS, and OS outcomes as shown in the univariate analysis, and patients with T1a/T1b disease had significantly better outcomes than those with T1c/T2a disease (Table 3). Although 90% of the patients with T1c/T2a disease achieved LC, six (17%) patients developed regional lymph node recurrence and/or distant metastasis (Table 2). Several studies demonstrated similar results in patients with T2 disease [20, 21]. Therefore, patients with T1c/T2 disease may require adjuvant treatments to prevent regional recurrence and/or distant metastasis. Several clinical studies are currently underway to evaluate the efficacy of adjuvant therapy using immune checkpoint inhibitors [22, 23].

Several studies showed that the incidence of grade 3 or higher radiation pneumonitis ranged from 2 to 9% in patients treated with Linac-SBRT or CK-SBRT [4, 5, 10–12, 24]. In the present study, only one (1%) patient developed grade 3 radiation pneumonitis. CK-SBRT enables highly concentrated dose distributions superior to those of Linac-SBRT. Several dosimetric studies have also suggested that CK-SBRT may have a dose concentration superior to that of Linac-SBRT [14, 25]. The tracking system of CK-SBRT also enables a reduction of the irradiated volume of the normal lung. These two aspects may have resulted in the low incidence of pulmonary toxicity in our study. In our patient population, the median V_20Gy_ was only 2.4% (Table 1).

In the current study, the 2-year PFS and LPFS were better in patients without than with a histological diagnosis (Table 3). Several other reports have also indicated that patients without a histological diagnosis had better treatment outcomes [26]. We made the utmost efforts to confirm the histological diagnosis for all patients; however, this was difficult in some cases.

The current study has several limitations. First, because it was a retrospective single-institutional study, selection bias is possible. Second, few patients developed local recurrence, which may have limited the statistical reliability. Third, this study included patients with clinical diagnoses, which may have impacted the treatment outcomes. However, CK-SBRT treatment planning was uniformly performed, and recording and reporting of the dose-volume histogram parameters were in strict compliance with published guidelines. Furthermore, almost all patients were strictly followed up. The present study therefore provides useful information on treatment. A long-term follow-up study is needed to confirm the efficacy and late toxicity of this treatment.

## Conclusions

High-dose CK-SBRT for stage I peripheral NSCLC produced favorable treatment outcomes with acceptable late toxicity. Further studies are needed to improve the treatment outcomes for patients with T1c/T2a disease.

## Data Availability

The datasets used and/or analyzed during the current study are available from the corresponding author on reasonable request.

## Abbreviations

BED: Biologically effective dose
CI: Confidence interval
CK: CyberKnife®
CT: Computed tomography
D_max_: Maximum dose
GTV: Gross tumor volume
ITV: Internal target volume
JCOG: Japan Clinical Oncology Group
LC: Local control
Linac-SBRT: Linear accelerator-based stereotactic body radiotherapy
LPFS: Local progression-free survival
NSCLC: Non-small cell lung cancer
OS: Overall survival
PFS: Progression-free survival
RTOG: Radiation Therapy Oncology Group
SBRT: Stereotactic body radiotherapy
V_20Gy_: Normal lung volume receiving more than 20 Gy
